# Experimental design approach in recombinant protein expression: determining medium composition and induction conditions for expression of pneumolysin from *Streptococcus pneumoniae* in *Escherichia coli* and preliminary purification process

**DOI:** 10.1186/1472-6750-14-1

**Published:** 2014-01-09

**Authors:** Guillermo Marini, Mateus Dalcin Luchese, Ana Paula Correa Argondizzo, Ana Carolina Magalhães Andrade de Góes, Ricardo Galler, Tito Lívio Moitinho Alves, Marco Alberto Medeiros, Ariane Leites Larentis

**Affiliations:** 1 Bio-Manguinhos (Instituto de Tecnologia em Imunobiológicos) - Fundação Oswaldo Cruz (FIOCRUZ) - VDTEC (Vice-Diretoria de Desenvolvimento Tecnológico), Av. Brasil 4365, Pavilhão Rockfeller Sala 202 - 21040-360, Manguinhos, Rio de Janeiro, RJ, Brazil; 2 Programa de Engenharia Química - COPPE - Universidade Federal do Rio de Janeiro (UFRJ), Av. Horácio Macedo 2030, Bloco G, Sala 115 - 21941-972, Ilha do Fundão, Rio de Janeiro, RJ, Brazil; 3 ENSP (Escola Nacional de Saúde Pública Sergio Arouca) - Fundação Oswaldo Cruz (FIOCRUZ) - Centro de Estudos da Saúde do Trabalhador e Ecologia Humana (CESTEH), Av. Leopoldo Bulhões 1480, Prédio 1º de Maio - 21041-210, Manguinhos, Rio de Janeiro, RJ, Brazil

**Keywords:** Soluble expression, Experimental design, Design of experiment (DoE), rPly, Recombinant *E. coli*, Hemolytic activity

## Abstract

**Background:**

*Streptococcus pneumoniae (S. pneumoniae)* causes several serious diseases including pneumonia, septicemia and meningitis. The World Health Organization estimates that streptococcal pneumonia is the cause of approximately 1.9 million deaths of children under five years of age each year. The large number of serotypes underlying the disease spectrum, which would be reflected in the high production cost of a commercial vaccine effective to protect against all of them and the higher level of amino acid sequence conservation as compared to polysaccharide structure, has prompted us to attempt to use conserved proteins for the development of a simpler vaccine. One of the most prominent proteins is pneumolysin (Ply), present in almost all the serotypes known at the moment, which shows an effective protection against *S. pneumoniae* infections.

**Results:**

We have cloned the pneumolysin gene from *S. pneumoniae* serotype 14 and studied the effects of eight variables related to medium composition and induction conditions on the soluble expression of rPly in *Escherichia coli* (*E. coli)* and a 2^8-4^ factorial design was applied. Statistical analysis was carried out to compare the conditions used to evaluate the expression of soluble pneumolysin; rPly activity was evaluated by hemolytic activity assay and served as the main response to evaluate the proper protein expression and folding. The optimized conditions, validated by the use of triplicates, include growth until an absorbance of 0.8 (measured at 600 nm) with 0.1 mM IPTG during 4 h at 25°C in a 5 g/L yeast extract, 5 g/L tryptone, 10 g/L NaCl, 1 g/L glucose medium, with addition of 30 μg/mL kanamycin.

**Conclusions:**

This experimental design methodology allowed the development of an adequate process condition to attain high levels (250 mg/L) of soluble expression of functional rPly in *E. coli*, which should contribute to reduce operational costs. It was possible to recover the protein in its active form with 75% homogeneity.

## Background

### *Streptococcus pneumoniae* and pneumococcal diseases

Since it was first isolated in 1881, *Streptococcus pneumoniae* is one of the most extensively studied microorganisms. In spite of the vast number of publications on the bacterium, many questions about its pathogenicity are still unanswered, and this pathogen remains a major causative agent of serious human diseases such as pneumonia, meningitis and bacteraemia [1]. Indeed, pneumonia is the leading cause of child mortality worldwide, with 1.9 million of the estimated 10 million child deaths each year, with meningitis as the second leading cause of child mortality. Additionally, pneumococcus causes noninvasive diseases such as acute otitis, sinusitis, conjunctivitis, and bronquitis. The incidence of pneumococcal diseases is raising and is a serious global problem. The vast majority of its victims come from the poorest countries in the world [2].

Differences observed in the chemical composition and also immunogenicity of the polysaccharide capsule (PS), make it possible to recognize more than 90 different serotypes of *S. pneumoniae* worldwide. Serotype prevalence may change based on patients age, geographical region and type of infection [3]*. S. pneumoniae* expresses a wide variety of surface proteins to interact with host cell components during the colonization or dissemination stages of the bacterium. They are also involved in the pathogenesis of the disease both as mediators of inflammation and as a direct part on host tissue attack [4].

### Pneumococcal vaccines and pneumolysin as a potential protein candidate for a new vaccine

Attempts to develop a vaccine to protect against the pneumococcus based on capsular polysaccharides yielded three licensed vaccines against *S. pneumoniae* which are commercially available: Pneumovax (Merck), Prevnar or Prevenar (Wyeth) and Synflorix (GSK). Pneumovax is a 23-valent PS vaccine with strong efficacy in adults but poor efficacy in infants and young children (<2 years) due to the lack of mature B cells. The other two available vaccines are based on PS conjugated to carrier proteins to enhance the immunologic response, with a broader efficacy in infants and young children, despite the poor protection against less common serotypes. The main disadvantages of these commercial vaccines are the high-cost production process, and the incomplete coverage of serotypes whose incidence may vary among countries [5,6].

Several pneumococcal proteins have been investigated in the past two decades, as an alternative to the expensive conjugate vaccines, as an antigen in potential protein-based vaccine candidates or as carriers for polysaccharides, aiming to broaden serotype coverage with less components in the vaccine [5-8]. In this regard, the most interesting proteins described to date are the pneumococcal toxin pneumolysin (Ply); choline-binding surface protein family (CBPs); pneumococal surface proteins, as the pneumococcal surface adhesin A (PsaA) and pneumococcal surface protein A (PspA); and the Caseinolytic protease (ClpP), among others under study. These proteins are present in the majority of pneumococcal serotypes isolated to date. Combinations of diverse pneumococcal proteins with conserved amino acid sequences may provide the best serotype-independent protection against *S. pneumoniae*.

Ply is a 471 amino acids monomeric protein with a molecular mass of 53 kDa, folded in four globular domains [9]. This protein shows a highly conserved 11 residues sequence in one of the domains, rich in tryptophan residues (ECTGLAWEWWR), suggested by earlier studies [10,11] as the responsible sequence for anchoring at host cell membrane and forming a transmembrane pore complex. Recent study by Soltani *et al.*[12] suggested that the cholesterol recognition motif resided within two structural loops, at the same domain where the 11 residues sequence are located. The pores upset the delicate osmotic balance between the cell and its environment, allowing material to leak in and out freely, quickly leading to lysis of the target cell [9,13,14]. Ply also presents other biological activities, depending on the concentration, especially at the initial stage of the pathogenesis of pneumococcal infection. In high concentrations, the enzyme is toxic to ciliated bronchial epithelial cells, reducing the ciliary movement, destroying the integrity of joints and cellular bronchial epithelial monolayer, facilitating the spread of pneumococcal infection. It also interacts with the epithelial cells of the alveoli and pulmonary endothelial cells, causing alveolar edema and hemorrhage during pneumococcal pneumonia, and easing the penetration from the epithelium to the pulmonary interstitium and ultimately into the bloodstream [14]. At low concentrations, this enzyme is able to inhibit effector functions (respiratory bursts) of neutrophils and monocytes, chemotaxis, bactericidal activity, and production of lymphokines and immunoglobulins [14]. Consequently, the phagocytic activity of the host cell is inhibited [9,15].

### Optimization of heterologous protein production

In order to express target proteins in high yields, recombinant DNA technology is the most appropriate tool and *Escherichia coli* is one of the most used hosts, due to its ability to grow rapidly at high cell density, with relative less expensive substrates, in addition to its well-established genetic background, and the existence of many commercial cloning vectors and strains suitable for expression [16-18]. An improvement in the process of expression of recombinant proteins can facilitate the subsequent step of purification. Consequently, achieving high expression levels of recombinant proteins is an essential step in the development of a bioprocess, towards maximum profitability and economic viability. However, there is no single, universal process for the expression of all recombinant proteins in this host, because the levels of expression of a heterologous gene will depend on multiple variables that are specific to each recombinant system. All these variables should be carefully evaluated because they influence the system [8,19].

The traditional method of process evaluation involves varying one variable at a time, while keeping other variables constant. This strategy requires a relatively large number of experiments and frequently fails to anticipate optimal conditions [20]. Changing one factor at a time does not depict the combined effect of all the variables involved. It is also a time consuming process because a large number of experiments is required. The deficiency can be overcome by applying more efficient, statistic-based experimental design. In this respect, factorial design is an important tool to determine optimal process conditions. The advantage of using the factorial design method is that many more variables can be screened simultaneously and much quantitative information can be extracted with a few experimental trials. Statistical methods through factorial experimental designs not only offer the simultaneous study of many variables, but also allow the study of interactive effects of many factors together, facilitating the prediction of the response for the values of variables not tested in the experiment [7,21]. In recent years, factorial designs - which are statistical techniques for designing experiments, defined significant effects, building models, evaluating the effects of variables and searching for optimum conditions - have successfully been used to optimize many bioprocesses [22,23]; however, they are not yet the most common strategies used to evaluate heterologous protein expression. The use of statistically designed experiments allows the rapid and economical determination of optimal culture conditions with fewer experiments and minimal resources [24]. Using a fraction of the complete factorial design is also advantageous in cases when the number of variables is larger than four, which demands a great number of experiments. The fraction of experiments should conserve the statistical condition of orthogonality that allows the estimation of independent parameters [22,25].

In view of these considerations, the aim of this study was to express recombinant Ply (rPly) in high concentrations and in its soluble form, using *E. coli* as a heterologous expression system. To improve the protein production, the many variables that influence the expression of this protein were evaluated, using experimental design. As such, fractional factorial design was used for eight variables related to medium composition and induction conditions. These variables are the concentrations of yeast extract, tryptone, glucose, glycerol, kanamycin, and inducer, the absorbance at induction moment, and the temperature of expression after the induction moment.

## Results and discussion

### Multivariant analysis and variables evaluated

The statistical experimental design methodology, where the response is evaluated by changing more than one variable at a time, allow the estimation of the variables that are statistically significant, taking into account the interactions between them. This multivariant method permits a thoroughly analysis compared to the traditional univariant method, where the response is evaluated changing one variable at a time while fixing the others. Furthermore, the multivariant method enables to characterize the experimental error, to compare the effects of variables between themselves when variables are normalized, and hence, to gather high-quality information with as few experiments as possible. All these advantages make this approach a powerful tool to optimize culture medium compositions and process conditions for recombinant protein expression [7,19,21,23,24,26-32].

When the recombinant protein is expressed intracellularly in host cells, it is known that to yield high amounts of that protein, it is necessary to achieve high cell growth. Thus, the higher the cell growth, the more recombinant protein is synthesized [33]. As a consequence, different operational strategies are developed to increase cell growth. However, it is also known that protein may be expressed in a soluble or insoluble form, into inclusion bodies. Several works in the literature consider the formation of these inclusion bodies as a drawback of the expression system in *E. coli*, because they require further addition of a complex process of isolation and purification, in which proteins are denatured and refolded *in vitro*. Therefore, the recovery of protein may be very low, which adds to the losses in all subsequent stages of purification, making the global recovery low [34]. Thus, a major challenge in recombinant expression is to design strategies for soluble production. To evaluate whether the protein expressed in a soluble form is directly related to cell growth, the first approach of this study was to determine the variables that have significant effects on cell growth and then compare that effect pattern with the one obtained for the protein expression, which in turn was evaluated through the pneumolysin biological activity assay.

In order to define a process with high yields and reduced time for the expression of the recombinant protein pneumolysin in its soluble form, we assessed the effects of eight variables related to medium composition and induction conditions on three relevant responses (cell growth, biological activity and productivity of rPly) in bench scale. To that effect, a statistical strategy based on a fractional factorial screening design was used, with two levels for each of the eight variables (2^8-4^) and replicas at the central point. A previous study of this expression system reported that induction times longer than 6 h were associated with lower productivity, meanwhile induction times between 4 h and 6 h presented similar levels of productivity [35]. Thus, for this system, the expression time was defined in 4 h, which means the highest level of productivity of pneumolysin, for the lowest operational time. A sequence of 24 different experimental conditions was run to gather information about these variables that may affect the rPly soluble expression. Results are presented in Table 1. When summiting the *E. coli* BL21 Star (DE3)™ strain with pET plasmid without the interest gene (negative control) to the same expression process as the *E. coli* BL21 Star (DE3)™/ pET28a/*ply,* no band enhancement was detected in SDS-PAGE analysis at the same size as rPly in the total protein extract after 4 h induction, and no biological activity was observed in the hemolytic assay.

**Table 1:** Fractional factorial screening design and responses

**Condition**	**Induction absorbance**	**[IPTG]**	**Expression temperature**	**[Yeast extract]**	**[Tryptone]**	**[Glucose]**	**[Glycerol]**	**[Kanamycin]**	**Cell growth**	**Hemolytic activity**	Process time (*)	**Productivity**
	***Abs***_**ind**_	***mM***	***°C***	***g/L***	***g/L***	***g/L***	***% v/v***	***μg/mL***	***Abs***	***HU/mL***	***min***	***HU/mL/min***
1	0.8 (-1)	0.1 (-1)	25 (-1)	5.0 (-1)	0 (-1)	1 (-1)	0.0 (-1)	10 (-1)	2.08	612	345	1.77
2	2.0 (+1)	0.1 (-1)	25 (-1)	5.0 (-1)	0 (-1)	10 (+1)	0.4 (+1)	50 (+1)	2.86	1171	450	2.60
3	0.8 (-1)	1.0 (+1)	25 (-1)	5.0 (-1)	10 (+1)	1 (-1)	0.4 (+1)	50 (+1)	2.62	1306	345	3.79
4	2.0 (+1)	1.0 (+1)	25 (-1)	5.0 (-1)	10 (+1)	10 (+1)	0.0 (-1)	10 (-1)	3.46	1306	425	3.07
5	0.8 (-1)	0.1 (-1)	37 (+1)	5.0 (-1)	10 (+1)	10 (+1)	0.4 (+1)	10 (-1)	3.18	678	340	1.99
6	2.0 (+1)	0.1 (-1)	37 (+1)	5.0 (-1)	10 (+1)	1 (-1)	0.0 (-1)	50 (+1)	6.42	1188	385	3.09
7	0.8 (-1)	1.0 (+1)	37 (+1)	5.0 (-1)	0 (-1)	10 (+1)	0.0 (-1)	50 (+1)	2.78	658	350	1.88
8	2.0 (+1)	1.0 (+1)	37 (+1)	5.0 (-1)	0 (-1)	1 (-1)	0.4 (+1)	10 (-1)	3.76	656	470	1.40
9	0.8 (-1)	0.1 (-1)	25 (-1)	23.6 (+1)	10 (+1)	10 (+1)	0.0 (-1)	50 (+1)	3.45	1446	345	4.19
10	2.0 (+1)	0.1 (-1)	25 (-1)	23.6 (+1)	10 (+1)	1 (-1)	0.4 (+1)	10 (-1)	4.73	1344	403	3.34
11	0.8 (-1)	1.0 (+1)	25 (-1)	23.6 (+1)	0 (-1)	10 (+1)	0.4 (+1)	10 (-1)	2.65	915	345	2.65
12	2.0 (+1)	1.0 (+1)	25 (-1)	23.6 (+1)	0 (-1)	1 (-1)	0.0 (-1)	50 (+1)	4.21	1146	420	2.73
13	0.8 (-1)	0.1 (-1)	37 (+1)	23.6 (+1)	0 (-1)	1 (-1)	0.4 (+1)	50 (+1)	4.09	639	335	1.91
14	2.0 (+1)	0.1 (-1)	37 (+1)	23.6 (+1)	0 (-1)	10 (+1)	0.0 (-1)	10 (-1)	5.47	1013	420	2.41
15	0.8 (-1)	1.0 (+1)	37 (+1)	23.6 (+1)	10 (+1)	1 (-1)	0.0 (-1)	10 (-1)	4.02	335	335	1.00
16	2.0 (+1)	1.0 (+1)	37 (+1)	23.6 (+1)	10 (+1)	10 (+1)	0.4 (+1)	50 (+1)	5.40	1353	420	3.22
17	1.4 (0)	0.55 (0)	31 (0)	14.3 (0)	5 (0)	5.5 (0)	0.2 (0)	30 (0)	3.60	1469	370	3.97
18	1.4 (0)	0.55 (0)	31 (0)	14.3 (0)	5 (0)	5.5 (0)	0.2 (0)	30 (0)	3.79	1557	370	4.21
19	1.4 (0)	0.55 (0)	31 (0)	14.3 (0)	5 (0)	5.5 (0)	0.2 (0)	30 (0)	3.73	1610	360	4.47
20	1.4 (0)	0.55 (0)	31 (0)	14.3 (0)	5 (0)	5.5 (0)	0.2 (0)	30 (0)	3.82	1615	350	4.61
21	1.4 (0)	0.55 (0)	31 (0)	14.3 (0)	5 (0)	5.5 (0)	0.2 (0)	30 (0)	3.82	1506	370	4.07
22	1.4 (0)	0.55 (0)	31 (0)	14.3 (0)	5 (0)	5.5 (0)	0.2 (0)	30 (0)	3.68	1507	370	4.07
23	1.4 (0)	0.55 (0)	31 (0)	14.3 (0)	5 (0)	5.5 (0)	0.2 (0)	30 (0)	3.24	1158	370	3.13
24	1.4 (0)	0.55 (0)	31 (0)	14.3 (0)	5 (0)	5.5 (0)	0.2 (0)	30 (0)	3.32	1263	370	3.41

(*)Process time refers to culture time from the inoculum growth at 37°C to the end of 4 h of induction at different conditions defined by the experimental design.

### Effect of variables on cell growth

After 4 h of induction, culture samples were taken to determine cell growth, measured at an absorbance of 600 nm. As shown in Table 1, cell growth was different among the 24 experimental runs, suggesting that certain variables, related to medium composition and induction conditions, have significant effects on that response. To determine which variables those are, a statistical study of the effects was carried out, considering significant effects with *p*-value lower than 0.1, as shown in Table 2.

**Table 2:** Effects of variables on cell growth, rPly activity and process productivity

	**Cell growth**		**rPly activity**		**Process productivity**	
	**Effect**	***p*-value**	**Effect**	***p*-value**	**Effect**	***p*-value**
Mean/interc.	**3.82**	**<0.0001**	**985.4**	**<0.0001**	**2.57**	**<0.0001**
Induction absorbance	**1.43**	**<0.0001**	**323.5**	**0.0016**	0.33	0.2248
IPTG	**-0.42**	**0.0387**	-52.0	0.5422	-0.19	0.4720
Expression temperature	**1.13**	**<0.0001**	**-340.8**	**0.0011**	**-0.91**	**0.0041**
Yeast extract	**0.86**	**0.0004**	77.0	0.3706	0.23	0.3930
Tryptone	**0.67**	**0.0027**	**268.2**	**0.0061**	**0.79**	**0.0095**
Glucose	**-0.33**	**0.0920**	**164.3**	**0.0685**	0.37	0.1797
Glycerol	-0.32	0.1011	44.8	0.5993	0.09	0.7241
Kanamycin	0.31	0.1163	**256.0**	**0.0082**	**0.72**	**0.0160**
Curvature	-0.40	0.2356	**950.5**	**<0.0001**	**2.85**	**<0.0001**

In bold variables with statistical significance with a confidence level of 90% (*p*-value < 0.1).

According to the statistical analysis (Table 2), six statistically significant variables could be recognized on the cell growth, since they showed *p-*values lower than 0.1: induction absorbance, inducer concentration, expression temperature, concentrations of yeast extract, tryptone, and glucose. The two other variables, concentrations of glycerol and kanamycin showed no significant effects. When four variables were considered (induction absorbance, expression temperature, and concentrations of yeast extract and tryptone), it was possible to observe positive effects on the response for higher values.

Based on the statistical analysis of cell growth, there were only linear effects, since curvature was not significant; that is, the highest cell growth could be achieved by defining the significant variables at their extreme levels depending on their positive or negative effects.

Cell concentration at induction is relevant, given the fact that the expression of heterologous proteins may inhibit the growth because of the metabolic burden implied in the expression process that restricts the energy supply for other cell processes. During the stationary phase, when cell growth reached its maximum, induction is not desired, since expression rate is proportional to the growth rate at the induction moment. Therefore, the best moment should happen when the metabolic activity is completely active and consequently the growth rate is peaking, which happens during the exponential growth phase [36]. Based on these facts, expression induction is done when culture is at the exponential phase, but depending on the induction system and the recombinant protein, expression could be done at the initial, intermediate or final part of that phase. For a high cell growth, induction is better in the middle of the exponential phase, according to the statistical evaluation.

In the case of inducer concentration, the effect on cell growth was negative, showing a lower response value as concentration is raised from the inferior to the superior IPTG level. This phenomenon could be explained by the toxic characteristic of IPTG, which is widely reported in literature [31,37]. Noteworthy, expression of other recombinant proteins from *S. pneumoniae* studied in our laboratory (PsaA and ClpP protease)*,* using this same host system, showed a negative effect of IPTG on cell growth [7,31].

Another variable with negative effect on cell growth was glucose concentration. This effect may be explained because of the medium acidification by glucose metabolization, which generates acidic by-products such as acetic acid. Hansen *et al.*[38] showed a direct correlation between acetic acid production and glucose consumption when the medium contains glucose. In contrast, when glucose was replaced for glycerol, no acetic acid was detected in the supernatant. However, in this study the only variable which was statistically significant for medium pH change was glucose concentration; meanwhile glycerol did not presented statistical effect.

On the other hand, a richer media in carbon source (yeast extract and tryptone) and the temperature enhanced cell growth but not necessarily the protein’s recombinant expression. Finally, kanamycin concentration showed no statistical effect on this response, as it happened when it was evaluated in recombinant ClpP protein expression [31].

### Effect of the variables on the pneumolysin biological activity and on process productivity

As it was previously discussed, heterologous protein expression in *E. coli* frequently happens in the cytoplasm. Consequently, to obtain high levels of the foreign protein, a high cell growth culture is necessary [33]. However, recombinant protein expression in high cell growth may happen in an insoluble and inactive way, forming inclusion bodies. Thus, to express biologically active recombinant proteins, such as enzymes, it is necessary to avoid inclusion bodies formation, which requires a thorough study of the medium composition and the expression conditions of the system. Processes in which protein was expressed in its active form were presented by Nikerel *et al.*[26,39], and Volontè *et al.*[29]. Those processes were optimized by using experimental design strategy.

The main disadvantage of expressing a recombinant protein in inclusion bodies is the high operational cost for its recovery in a soluble way when it is needed [33,40]. From an operational point of view, the importance of a correct expression of a heterologous protein is to go directly to the purification step, avoiding additional previous steps, such as solubilization, oxidation, and re-folding, during which a great amount of material is lost [34].

In this study, the aim was to define a process to express rPly in its soluble form. As previously discussed, it was necessary to evaluate the soluble fraction of pneumolysin, which is the active fraction, expressed in all 24 different operational conditions, to define the most appropriate expression condition and then compare it with the condition for the best cell growth to determine whether the soluble rPly expression was correlated to cell growth.

The soluble fraction of the protein was assessed by measuring its hemolytic activity, since this is the only way to measure the exact and correct structural folded protein [41]. The statistical analysis of the effect of the eight variables on the biological rPly activity is presented in Table 2.

According to results in Table 2, five variables presented statistically significant effects on rPly activity: induction absorbance, expression temperature, and the concentrations of tryptone, glucose and kanamycin, since they presented *p*-values lower than 0.1. The curvature was also statistically significant, which means some responses do not have linear behavior, consequently, the best response corresponds to an intermediate value of the variable within the range tested, in contrast with linear responses that were found at the extreme levels of the range tested.

The negative effect of expression temperature was the main effect on rPly activity, since it was the highest value, and is in accordance with other studies, which also observed that lower temperatures reduced the rate of protein expression, thus avoiding the formation of inclusion bodies, enhancing soluble expression [28,29]. Volontè *et al.*[29] and Larentis *et al.*[7] showed that there was growth rate reduction when expression temperature was decreased from 37°C to 25°C, with a concomitant enhancement in the soluble protein quantity expressed. They also recognized that a lower reduction of the expression temperature implies an additional cooling step, making it a more expensive process, besides promoting significant reduction of cell growth. A similar result was found by Swalley *et al.*[21], when they studied medium composition and induction conditions to express a viral protein in *E. coli*. However, depending on the protein, it may be adequate to reduce the temperature to enhance the expression, and, consequently, productivity [7].

Concerning the induction absorbance, to achieve high amount of rPly in its soluble form, induction at high cell concentration is preferable. The concentrations of tryptone and glucose also had positive effect on rPly activity as well as kanamycin concentration. This kanamycin effect can be explained due to its contribution to the plasmid stability, maintaining high concentration of plasmid bearing cells, favoring the expression. At the lowest kanamycin concentration, probably, the plasmid segregation was drastically higher than that at higher antibiotics concentration. Based on a previous study carried out in our laboratory, it is known that plasmid segregation occurs in the same pET expression system for ClpP protease production, even at the highest kanamycin concentration evaluated in this study [31]. However, as the curvature is significant, and the highest activity was observed at the central point, the best condition to achieve a high activity could be defined at middle concentrations of these variables (tryptone, glucose and kanamycin concentrations).

The other three variables, concentrations of IPTG, yeast extract and glycerol, have no statistically significant effects (*p* > 0.1). The lack of statistical effect leads these variables to be defined at their lowest values to reduce operational costs. Therefore, inducer concentration may be reduced 10-fold compared with the traditional IPTG concentration used in most expression systems [26,29,34,36]. That is an advantage because of its toxic effect on cell growth, as discussed before. This was also observed for the IPTG-based expression of PsaA and ClpP proteins (within the 0.1 mM to 1 mM range) in the same cell host studied in our laboratory [7,31]. Yeast extract concentration can be fixed at 5 g/L, which is the lowest value normally used in most studies [26,34]. Glycerol concentration was evaluated as a glucose substitute to avoid acetate formation and to favor protein stability [38], but it showed no beneficial effects on rPly expression.

Comparing the patterns of the significant effects on the cell growth and on the biological activity, we could say there is no direct relationship between the solubility of rPly and cell growth. Thus, to define the medium composition and the induction conditions of a process for the soluble expression of rPly, the activity is the main response to be considered. However, the 24 different processes presented a great variability in terms of process times because they were induced at an absorbance of 0.8, 1.4, and 2.0. Therefore, in order to avoid erroneous conclusions, the statistical analysis should be done taking into account the biological activity regarding the total process times, that is, the statistical analysis should be done considering the different process productivities, as it is shown in Table 2.

The results presented in Table 2 show that expression temperature was the main variable affecting productivity, as observed on the biological activity analysis. The negative effect means the highest productivity level was reached at lower temperatures within the range tested. The concentrations of tryptone and kanamycin also significantly affected process productivity, but in contrast with the expression temperature, their effects were positive, which means that, for higher productivity levels, these variables should be defined at higher levels. On the optimization process for the rPsaA expression, tryptone did not show statistical effect and thus could be possibly removed from the medium [32]. Once more, the curvature was also statistically significant, conferring a non-linear effect to one or more variables. As curvature was positive, productivity peaks within the evaluated range, rather than at the extremes of that range. Therefore, to define the best medium composition and induction conditions, it is important to consider the productivity levels at the central point, where the response presented the highest level. In contrast, the other variables did not have statistical effects on the process productivity. These variables should be defined at their lowest levels within the tested range, to reduce process costs as much as possible without interfering on the productivity level of the process.

Based on the previous discussion and on the factorial design analysis, it is possible to infer the more accurate condition which allows the maximization of system productivity. Therefore, the medium composition was defined as follows: 5 g/L yeast extract, 5 g/L tryptone, 1 g/L glucose, 30 μg/mL kanamycin, without glycerol, and with 10 g/L NaCl (variable not evaluated in this factorial design). The expression condition was defined by inducing the expression at absorbance of 0.8, with 0.1 mM IPTG, and allowing for 4 h induction at 25°C.

The defined process showed that it is not necessary to use a culture medium richer in yeast extract, tryptone, or glucose, as most systems to express recombinant proteins normally use. In this medium, yeast extract content can be reduced about 5-fold compared to TB, a richer medium used in *E. coli* cultures [32]. Tryptone and kanamycin can also be reduced 2-fold in comparison to the highest concentrations tested, associated with TB and LB media content, and referred in Sambrook and Russell [42] molecular cloning laboratory manual, respectively; inducer, which is an expensive component, can be reduced 10-fold compared with the standard concentration used to induce this kind of system [42], and it is not necessary to add glycerol. Regarding expression temperature, its reduction to 25°C leads to energy saving, meanwhile induction at absorbance 0.8 means shorter process time.

The choice of tryptone and kanamycin concentration levels was due to their significant and positive effects in experiments, being that the highest productivity levels were obtained when these variables were set at their central levels. In addition, as the curvature was significant and positive, both concentrations, statistically significant, leading to a higher response when they are defined at the central levels of the ranges tested. Once the medium composition and induction conditions were defined according to the statistical analysis on the productivity of pneumolysin, the next step was to validate that process.

### Validation of the process defined by factorial design

Validation of the process defined by the factorial design was carried out in triplicate during 4 h kinetics (k1, k2, k3). Cultures in the medium grew at 37°C until reaching exponential growth phase (*Abs*_ind_ 0.8), when they were induced with 0.1 mM IPTG and temperature was reduced at 25°C to express the recombinant pneumolysin. After that, cell culture samples were taken every hour until the end of expression process (4 h). These samples were used to evaluate cell growth and productivity through process time. Results are shown in Table 3.

**Table 3:** Cell growth and productivity for the validation experiment

**Induction time**	**rPly activity**	**Cell growth**	**Yield per cell**
***h***	***HU/mL***	***Abs***	***HU/mL/Abs***
0*	0	0.82 ± 0.01	0
1	401 ± 18	1.60 ± 0.04	251 ± 7
2	656 ± 62	2.42 ± 0.09	272 ± 33
3	867 ± 20	2.65 ± 0.02	328 ± 6
4	1394 ± 73	2.93 ± 0.17	476 ± 34

Results are averages of three experiments. Expression of rPly was performed at *Abs*_ind_ 0.8 with 0.1 mM IPTG for 4 h induction at 25°C in a medium culture comprised of 5 g/L yeast extract, 5 g/L tryptone, 1 g/L glucose, 30 μg/mL kanamycin and 10 g/L NaCl.

*Induction moment.

The average of the pneumolysin activity in the validation condition was 1394 ± 73 HU/mL, which is statistically the same as the highest values obtained in the fractional design experiments. The total process time was 310 min (growth phase of 70 min and expression phase of 240 min), which is the minimum process time obtained in the factorial experiments. Thus, it can be observed that in this validated process, in which most of the medium composition and induction variables were defined at their lowest values, the pneumolysin soluble expression was high even though the medium composition was poorer and the total process time was the shortest.

**Figure 1:**
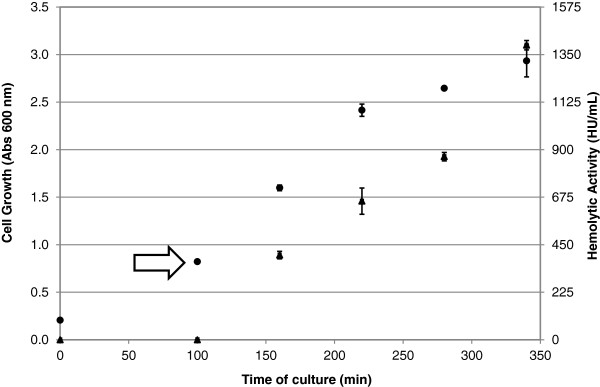
**Cell Growth (●) and rPly Activity (▲) through the total process.** Arrow is indicating the induction moment. Standard deviation of triplicate runs.

Figure 1 shows cell growth and the hemolytic activity of the soluble fraction of the pneumolysin, through the total process time. It is important to highlight that there was no hemolytic activity detected in the strain *E. coli* BL21 Star (DE3)™/pET28a culture (negative control), in the same culture and expression conditions of the strain *E. coli* BL21 Star (DE3)™/pET28a/*ply*. Thus, the hemolytic activity is related to the recombinant protein pneumolysin.

The cell growth kinetic show that after a two-hour induction (190 minutes of process), the cells started to reach the stationary growth phase, while the rPly activity was still rising. As discussed above, the pneumolysin expression, measured through its hemolytic activity, is not directly associated with cell growth, which can be confirmed in this kinetics (Figure 1). Based on the literature, this might happen due to the use of systems with strong promoters, such as T7 promoter. This kind of promoter, during induction, makes the cell energy turn to the protein expression to the detriment of cell growth, consequently the growth rate drops because the host cell metabolism is overburdened [31,43]. The specific growth rate obtained in this study was 0.56 h^-1^. Possible differences with other recombinant *E. coli* may be related to the different gene expressed, the interaction host/vector as well as the properties of the expressed protein [34,40].

The productivity average obtained in the validated process defined from the statistical fractional design was 4.50 HU/mL/min, with a standard deviation of 0.24 and a relative standard deviation of 5.3%. Comparing this value with the ones of the fractional design experiments, it is statistically equivalent to those obtained in experiment 9 (4.19 HU/mL/min), as well as those experiments performed at the central point (3.99 ± 0.50 HU/mL/min). These values are the highest productivity values obtained among the values from design experiments. That is relevant since highest rPly productivity levels were noticed in a relative low-cost and time-consumption.

### Preliminary purification process

The characteristics of recombinant protein production, the molecule location, and protein destination determine its purification process. Within the biopharmaceutical production processes, purification accounts for a high part of product cost, because it often results in low recovery of protein.

**Figure 2:**
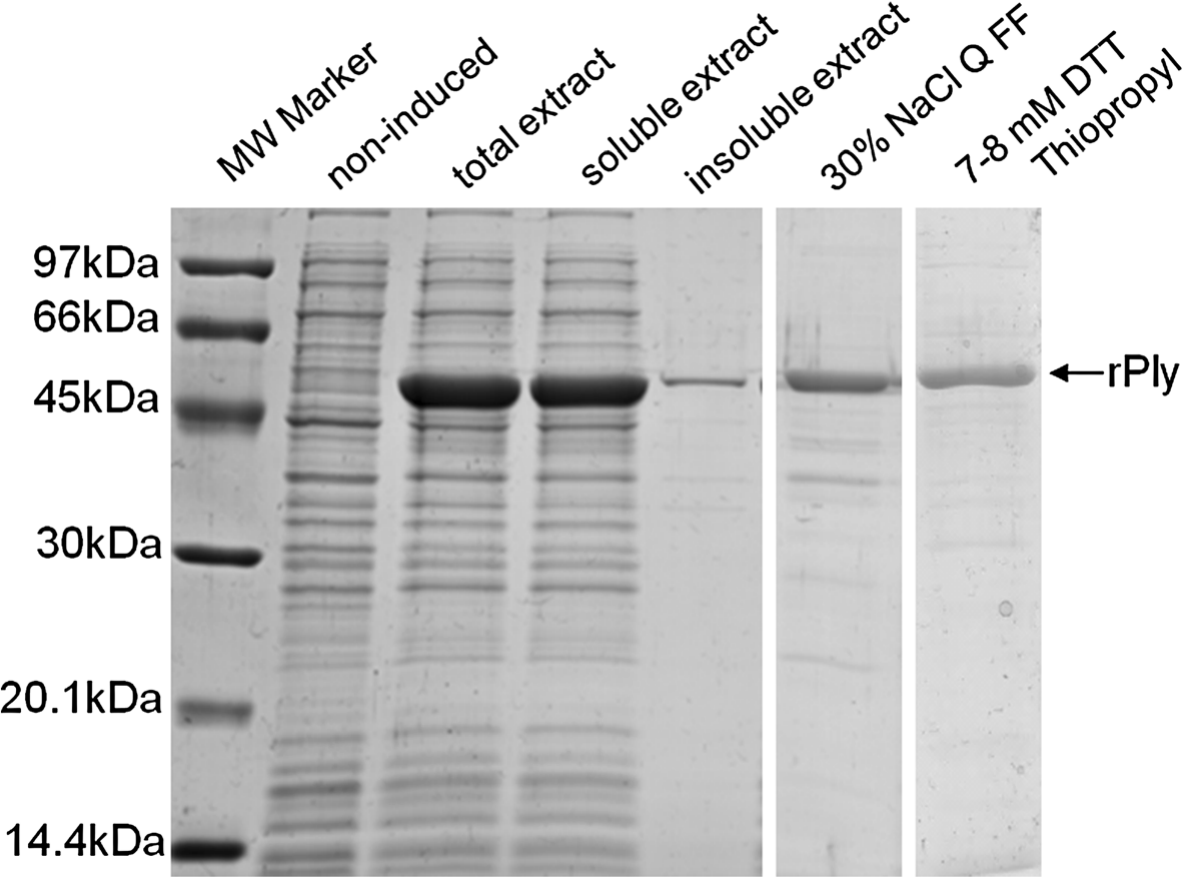
**SDS-PAGE of rPly expression and preliminary purification.** Non-induced sample and rPly expression at 25°C in TB medium after 4 h induction (total protein extract, soluble and insoluble fractions). Preliminary purification fractions obtained with 30% NaCl elution using Q FF column and 7-8 mM DTT elution using Thiopropyl matrix (MW = molecular weight).

In this work, the soluble fraction of rPly expressed in *E. coli* at 25ºC was used at the preliminary purification tests. The expression of rPly in total extract, soluble and insoluble fractions are presented in Figure 2. The densitometry analysis of the rPly band performed on the SDS-PAGE indicated that this protein is expressed around 250 mg/L, which corresponded to 25-28% of the total *E. coli* protein extract. The soluble fraction of rPly was around 90% from the total of rPly expressed in this condition.

Recombinant protein purification was conducted using two different strategies, ion exchange and affinity chromatography, without fusion tags. These strategies were run separately. The preliminary purification results of both strategies are also presented in Figure 2, with the recovery of a 75% homogeneity rPly fraction (shown in SDS-PAGE). Considering there are not many works in literature performing purification of rPly, and to our knowledge, neither Q FF column nor Thiopropyl Sepharose column were tested for this protein, these preliminary results could be considered as prominent. However, both methods need to be optimized in order to enhance recovery yields, as well as homogeneity, and to develop a feasible purification method that could be used in a production process of rPly. Hemolytic activity of the protein was confirmed after both processes, which was maintained after 8 days stored at 4ºC. These results for one-step purification of rPly without tags were comparable to those homogeneities described in the literature for several purification steps [44], and allowed the production of the recombinant protein with conformation closer to the original fold [7]. One of the major advantages of our cloning strategy without tags for purification is its ability to produce a protein which is ready to be used in biopharmaceutical applications, eliminating the tag removal step after purification, which is a step that increases process costs.

## Conclusions

Different levels of a soluble form of recombinant pneumolysin were obtained in all the experiments of the fractional design used. As there was no direct relationship between cell growth and soluble rPly expressed, and as total process time varied in the different levels of absorbance at induction, biological activity was the main response to be considered to find out the variables with statistically significant effects on productivity. The temperature of expression and the concentrations of tryptone and kanamycin were the three variables which affected productivity. The defined process was successfully validated, which yielded 4.50 HU/mL/min, one of the highest productivity values obtained compared with the productivities of the experimental design and with reduced operational costs. It should, therefore, be possible to reduce the inducer concentration ten-fold, reduce absorbance time of the induction moment and the concentrations of yeast extract, glucose, and glycerol to the minimum levels of the ranges tested, keeping rPly protein expression at high levels (250 mg/L, which corresponded to 25-28% of the total *E. coli* protein extract) and reducing process costs.

Once more, and in accordance with different literature data, the results presented emphasize the importance of experimental design to improve the biotechnological conditions of processes used to express a recombinant protein in *E. coli*. It was also proven it is necessary to have a minimum number of experiments to gather relevant information on the effect of each of the variables under study, as well as the experimental error. In contrast with traditional univariant methods, the assessment of various variables at the same time avoids the lack of considerations about the interactions of the relevant effects. All these advantages make experimental design a powerful tool for defining an optimized recombinant protein production process.

The reduction in the production cost of this protein - as well as other potential target proteins used in a possible protein-based vaccine, through the use of experimental design - demonstrated how essential this statistical tool is to obtain a highly efficient commercial vaccine with low-cost production to be accessible to the world’s most underprivileged populations.

## Methods

### Chemicals

Bacto™ yeast extract and tryptone were purchased from BD (Becton, Dickinson and Company), glucose and NaCl were purchased from Merck, glycerol from Invitrogen, kanamycin from Sigma and IPTG (isopropyl β-D-1-thiogalactopyranoside) was purchased from Promega. Potassium salts (K_2_HPO_4_ and KH_2_PO_4_) used in the medium were purchased from Merck.

### Cloning procedure

The *Escherichia coli* BL21 Star (DE3)™ strain (Invitrogen) was used as a host for the plasmid pET28a (Novagen) harboring the gene responsible for pneumolysin expression, and kanamycin resistance. Pneumolysin protein-coding gene was isolated from *S. pneumoniae* serotype 14, since it is the most prevalent in Brazil and also one of the most prevalent and virulent serotype in the world. The clone expressing the recombinant protein pneumolysin was developed at LATER/Bio-Manguinhos/FIOCRUZ and cloning techniques were reported in Larentis *et al.*[7] and Einsfeldt *et al.*[31]. The clone cryo-preservation was at -70°C in 25% glycerol (Invitrogen). The working vials of the clone *E. coli* BL21 Star (DE3)™/ pET28a/*ply,* thawed from the working bank, were evaluated in reference to their cell viability.

### Cell viability test

The cell viability of the working vials of recombinant *E. coli* BL21 Star (DE3)™/pET28a/*ply* in LB (5 g/L yeast extract, 10 g/L tryptone, 5 g/L NaCl, pH 7.0) with 25% glycerol, stored at -70°C, was assessed by counting the colony forming units (CFU) for all the experimental design experiments. Serial dilutions were made in PBS pH 7.4 and transferred to Petri plates containing LB Agar and 50 μg/mL kanamycin (concentration of working vials were, on average, 28×10^10^ CFU/mL).

### Variables and experimental design

Eight variables of the expression process, related to both the composition of the culture medium and the induction conditions, were chosen to be evaluated by the experimental design: concentrations of yeast extract, tryptone, glucose, glycerol, kanamycin and inducer, absorbance at induction moment, and expression temperature (Table 4).

**Table 4:** Variables and levels assayed

	**Inferior level**	**Central level**	**Superior level**
	**(-1)**	**(0)**	**(+1)**
Induction absorbance (*Abs*_ind_)	0.8	1.4	2.0
IPTG (mM)	0.1	0.55	1.0
Expression temperature (°C)	25	31	37
Yeast extract (g/L)	5	14.3	23.6
Tryptone (g/L)	0	5	10
Glucose (g/L)	1	5.5	10
Glycerol (%v/v)	0	0.2	0.4
Kanamycin (μg/mL)	10	30	50

To evaluate the statistical effects of the variables on the soluble expression of pneumolysin, a fractional factorial design 2^8-4^ was chosen, with replicates on the central point. This fractional factorial design is resolution IV, which means that the main effects can be determined, but not their interactions [22]. The curvature was also evaluated by the factorial design, which allowed us to learn whether effects are linear or not. When a variable effect is linear, the highest response is obtained at the highest level of the variable for a positive effect, or at the lowest level for a negative effect. On the other hand, when a variable effect is not linear, the highest or the lowest response values could be obtained at an intermediate level of the variable within the range evaluated.

Due to the large variability inherent to bioprocesses, the parameters were considered significant with *p*-values lower than 10% (*p* < 0.1). Using 90% confidence in the statistical analysis (Software *Statistica* 9.1/Statsoft) allowed the inclusion of possible relevant variables that would be excluded from a strict statistical analysis with a significance level of 5% (*p* < 0.05). The normalization of the levels of each variable allows the comparison of the importance of each variable effect, in reference to the evaluated response. Once the most adequate experimental design was chosen, the next step was to select the range of values to be studied for each of the eight variables, defining the lowest level as -1, the highest level as +1, and 0 as the central point calculated as the middle of these two levels (Table 4). IPTG and kanamycin levels were defined based on information found in literature [35,42]; induction conditions and media composition were chosen according to previous studies with pneumolysin [35] and other *S. pneumoniae* proteins expressed in *E. coli*[7,31,32]; temperature levels were selected evaluating rPly solubility [35]; induction absorbances were defined at the initial (absorbance 0.8 measured at 600 nm) and at the middle (absorbances 1.4 and 2.0) exponential phase from cell growth kinetics in different medium compositions and temperatures [35]; media components levels were chosen based on LB (Luria-Bertani) and TB (Terrific Broth) media compositions [32,35].

### Protein expression and purification process

Recombinant *E. coli* BL21 Star (DE3)™/pET28a/*ply* was pre-inoculated (10 μL of working vial) in 10 mL of the TB medium (23.6 g/L yeast extract, 11.8 g/L tryptone, 9.4 g/L K_2_HPO_4_, 2.2 g/L KH_2_PO_4_, pH 7.2) enriched with 1% glucose, 0.4% glycerol and 50 μg/mL kanamycin. The pre-inoculum was incubated for 16 hours at 37°C and 200 rpm in 50 mL flasks under agitation; simultaneously, the cell viability test was done. After 16 h, the 24 different expression experiments were done in 6 different sets of experiments and in each group a central point repetition was performed, as discussed below. The inoculum was prepared in 500 mL flasks with 2 mL pre-inoculum and 100 mL of the medium, with the corresponding yeast extract, tryptone, glucose, glycerol, and kanamycin concentrations, according to the experimental design (as described in the previous section). The culture was incubated at 37°C and 200 rpm until it reached the evaluated values of the exponential growth phase. At this point, expression was induced with the corresponding IPTG concentration for four hours under different expression temperature, as the experimental design indicated. In addition to the 16 different expression conditions, 8 replicates of the central point were done to define the experimental error associated to this kind of expression process, as well as the curvature, resulting in the 24 different runs. Samples of 1 mL volume were taken from each experiment before and after the four-hour expression period to assess cell growth and soluble rPly expression (by hemolytic activity).

The same expression process was done using an *E. coli* BL21 Star (DE3)™ strain, with the pET28a plasmid without the interest gene, as a negative control, to confirm the lack of expression of rPly in a SDS-PAGE analysis and also the lack of biological activity in an hemolytic analysis.

The recombinant protein was purified in an ÄKTA Purifier 10 system by strong anion exchange in a 1 mL Q FF column (Hitrap Q Sepharose™ Fast Flow/GE Healthcare) with 50-100 mM NaCl added to the initial sample and eluted with 20 mM Tris HCl and 300 mM NaCl (pH 8.0). Purification was also tested by affinity using a Thiopropyl Sepharose™ 6B matrix (GE Healthcare) that reacts with solutes containing thiol groups, and elutes using the same elution buffer with 5-25 mM DTT.

Samples were analyzed by 12.5% SDS-PAGE stained with Coomassie blue and densitometry was used to evaluate the concentration related to the rPly band in the total protein extract, soluble and insoluble (inclusion bodies) fractions and also to evaluate the homogeneity of purification fractions in a Bio-Rad GS-800 Calibrated Densitometer/QuantiOne 4.4.1 software according to Larentis *et al.*[7].

### rPly solubility by hemolytic activity measurements

The cells from the pre-induction (0 h) and after 4 h induction samples were harvested by centrifugation at 20817 × *g* for 10 min at 4°C to separate the culture medium. In order to solubilize the expressed rPly, the cells were resuspended in a lysis buffer (20 mM Tris, 1 mM EDTA, 200 mM NaCl, pH 8.0) restituting the 1 mL sample volume, to obtain the total protein extract. The total extract was disrupted by eight 10-second ultrasound cycles at 60% amplitude in an ultrasonic cell disruptor (Sonics & Materials, Inc.). The soluble and insoluble protein fractions were separated from the cultures by centrifugation (20817 *g* for 10 min at 4°C). The soluble fraction was used to assess the hemolytic activity.

In round-bottomed 96-well microplates, a two-fold serial dilution was done with 100 μL of soluble fraction from each experimental condition using the lysis buffer (20 mM Tris, 1 mM EDTA, 200 mM NaCl, pH 8.0). A volume of 100 μL of triple-washed 2% defibrinated bovine blood cells in the same lysis buffer was added to the dilutions and incubated for 1 h at 37°C and overnight at 4°C, so as to allow unlysed red blood cells to sink to the bottom of the well and form a clear red dot in the transparent solution. On the other hand, lysed cells release their hemoglobin, giving the solution a red color that allows the monitoring of protein lysing activity. A volume of 100 μL supernatant from each well was transferred to a new flat-bottomed 96-well microplate. The hemolytic activity was evaluated spectrophotometrically at 540 nm by assaying the hemoglobin released in the supernatant. Samples with 2% blood cells completely lysed as a 100% lysis reference were serially two-fold diluted in lysis buffer to produce the standard curve; the supernatant of 2% erythrocyte suspension (100 μL) incubated with lysis buffer was used as the blank reference (0% lysis). One Hemolytic Unit (HU) was expressed as the quantity of rPly presented in a sample comparing with the standard curve, which allows the obtainment of complete hemolysis of a 2% erythrocyte suspension, after 1 h incubation at 37°C. Therefore, the first well of the standard curve contained 1 HU. That hemolytic activity was related to 1 mL of sample.

## Competing interests

The authors declare that there were no competing interests in this study.

## Authors’ contributions

GM carried out all the experiments for the evaluation of the statistical influence of medium composition and induction conditions for the soluble expression of the recombinant Ply in *E. coli*, performed the hemolytic activity measurements, the statistical analysis and the preparation of the manuscript. MDL carried out the experiments to define the levels of variables and the duration of induction time based on system productivity and revision of the manuscript. APCA carried out the cloning of the recombinant protein in the host cell and revised the manuscript. ACMAG participated in the purification strategy and experiments. RG made periodic analysis of the project for the development of a protein vaccine against pneumonia and careful revision of the manuscript draft. TLMA participated in the design of the study, discussion and interpretation of the bioprocess and hemolytic activity data. MAM was responsible for bioinformatics analysis and cloning strategy, and also participated in the discussion of the data, coordinated financial support from LATER/Bio-Manguinhos/Fiocruz and revised the manuscript. ALL was responsible for the project and student coordination, as well as for the design of the statistical study, discussion and interpretation of the statistical analysis, for the financial support from PAPES/Fiocruz and helped to draft the manuscript. All authors have approved the manuscript.
